# Portraits of the Skin

**Published:** 1849-07

**Authors:** 


### Bibliographical Notices.

*Portraits of the Skin.* By Erasmus Wilson, F. R. S. Fasciculus. Four Plates. London, Churchill, folio.

The author of this beautiful work is the consulting surgeon to St. Pancras Infirmary, and has devoted many years to the investigation of diseases of the skin which has attained for him a European reputation. The work before us is purely practical, and if Mr. Wilson only completes his undertaking in the same manner as begun, it will do honor not only to British medicine, but will secure to him the gratitude and respect of every nation where the healing art is practiced.

The author has so well explained his intentions in his preface, that we cannot do better than transcribe his own words in this notice.

"In selecting a title for the present work, I have been actuated by a desire of representing in a word my intentions with regard to its objects. I have determined that no labor or expense shall be spared to render it complete as a series of 'Portraits of Diseases of the Skin.'

"In another sense, I might have termed the work 'Illustrated cases of cutaneous disease,' inasmuch as it is my intention to report the case of the patient with each plate, introducing among the details of the case such general deductions and practical observations as may tend to explain more fully the history and management of the disease. By following this plan, the student will be placed in the position of the practitioner before whom the case is brought, and the peculiar characters of the disease being thus strongly impressed on his mind, will be made available when a similar case is presented to his notice in practice.

"Indeed, the cases will be given, and the portraits drawn, in the order in which they come before me, and without reference to classification; the circumstance determining my selection being the character of the case as a good specimen of the particular disease, and the instruction which it may be calculated to convey. By pursuing this course, the practitioner will have ensured to him the most characteristic and best examples of cutaneous disorders, and the author will be relieved from the yoke of following a particular arrangement in the sequence of his illustrations.

"Another advantage will arise out of this plan, which is, that the artist, by transferring his drawing immediately to the stone, will be enabled to complete his coloring of the lithograph from the patient, and while all the peculiar tints of the disease are fresh in his mind."

As to the completion of the work, he observes "it will be my endeavor to complete, as soon as possible, the entire series of cutaneous diseases, in order to form a volume for binding. When I have affected this object, I shall supply a table by which the plates may be arranged according to a classified order."

